# Supplementary material for: Radiative cooling and indoor light management enabled by a transparent and self-cleaning polymer-based metamaterial
Source: Nat Commun. 2024 May 7;15:3798. doi: 10.1038/s41467-024-48150-2 (PMC11076518; doi:10.1038/s41467-024-48150-2)
Supplement: Supplementary file 1 — Supplementary Information [file 41467_2024_48150_MOESM1_ESM.pdf]

Radiative cooling and indoor light management enabled by a transparent and self-cleaning polymer-based metamaterial

Gan Huang[1,*], Ashok R. Yengannagari[1], Kishin Matsumori[1], Prit Patel[1], Anurag Datla[1], Karina Trindade[1], Enkhlen Amarsanaa[1], Tonghan Zhao[1], Uwe Köhler[1], Dmitry Busko[1], Bryce S. Richards[1,2,*]

[1] Institute of Microstructure Technology, Karlsruhe Institute of Technology, Hermann-von-Helmholtz-Platz 1, 76344 Eggenstein-Leopoldshafen, Germany.

[2] Light Technology Institute, Karlsruhe Institute of Technology, Engesserstrasse 13, 76131 Karlsruhe, Germany.

* Corresponding authors: gan.huang@kit.edu; bryce.richards@kit.edu

Supplementary figures

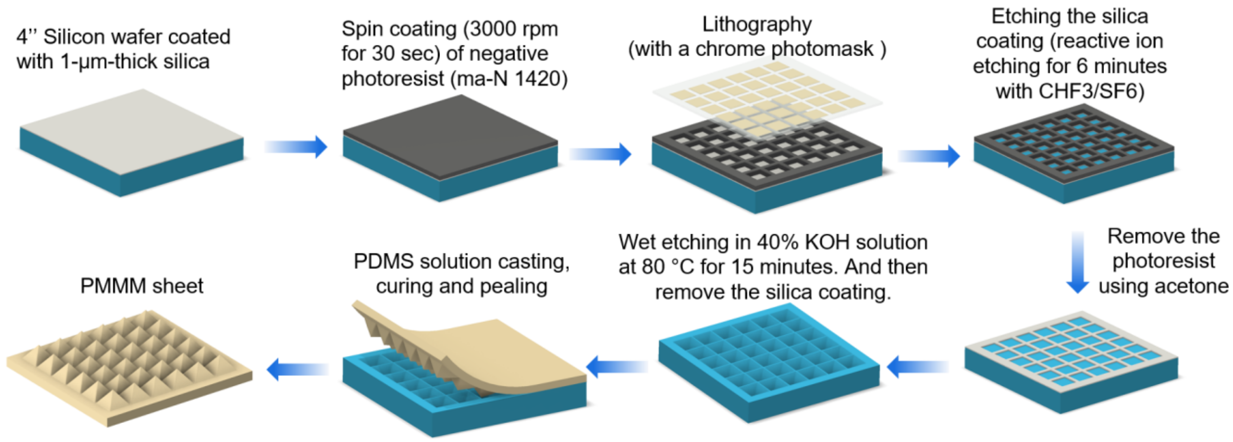

Supplementary Fig. 1. Detailed PMMM fabrication process. A 4-inch (100)-oriented polished silicon wafer coated with 1-µm-thick silica was selected as the base wafer for the mould fabrication. Negative photoresist (ma-N 1420) was then coated on the silicon wafer by spin coating (3000 rpm for 30 seconds). The 5-inch chrome photomasks with the designed patterns (fabricated by JD Photo Data, the UK) were used in a photolithographic setup via a mask aligner (EVG620) at 365nm wavelength. The 1-µm-thick silica coating was selectively etched by reactive ion etching for 6 minutes with CHF3/SF6, and then the photoresist was removed by acetone. The silicon wafer was then soaked in 40% KOH solution at 80 °C for 15 minutes for anisotropic wet etching (etching rate: ~75 µm/h), to reveal the (111) crystal planes that serve as the faces for the inverted micro-pyramids. Anti-adhesive coating synthesis: 20 µL trichloro-(1H,1H,2H,2H-perfluoroctyl)-silane (PFOTS, VWR) was mixed with 10 mL cyclohexane (VWR). The PFOTS solution is then coated on the silicon wafer for 1 hour via evaporation coating. Polydimethylsiloxane solution casting: the base silicone elastomer and curing agent (VWR) was mixed with a ratio of 10:1 and stirred for 10 minutes. The mixture was then put in a vacuum oven for more than 1 hour until all the bubbles in the mixture were removed. The polydimethylsiloxane mixture solution was pasted onto the silicon wafer via blade coating. The silica wafer was then heated by a hot plate heater at 90 °C for 15 minutes. The cured PMMM film was peeled off from the silicon wafer manually.

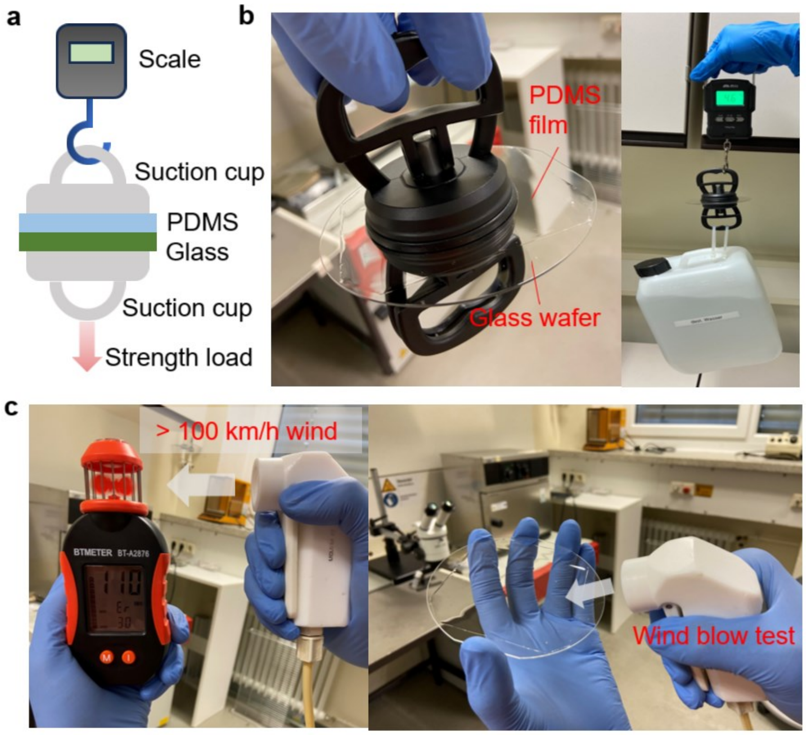

Supplementary Fig. 2. Adhesion strength testing. a, Schematic of the adhesion strength testing. The PDMS-glass interface of the aged sample is subjected to a load using two suction cups to assess its adhesive strength. **b**, We fabricated a sample by applying a PDMS coating onto a 6-inch glass substrate. In this study, the PMMM is made of polydimethylsiloxane (PDMS). PDMS naturally exhibits excellent adhesion to glass, a property attributed to the interactions between its siloxane groups (Si−O−Si) and the silanol groups (Si–O–H) present on the glass surface[1]. Consequently, additional adhesive is not necessary. Subsequently, this sample was placed outdoors for a duration of two months at the North Campus of the Karlsruhe Institute of Technology in Karlsruhe, Germany. The PDMS-glass construct can bear a pressure larger than 4 kPa (~4.6 kg load on 0.0098 m^2, as indicated in the figure). Owing to PDMS's renowned stability, the bond it forms with glass remains steadfast, especially when appropriately cured. **c**, Wind blowing testing. We used compressed gas to simulate the strong wind in outdoor applications. The compressed gas generated >100 km/h speed wind (close to the violent storm). The PDMS film on the glass can easily withstand the simulated strong wind.

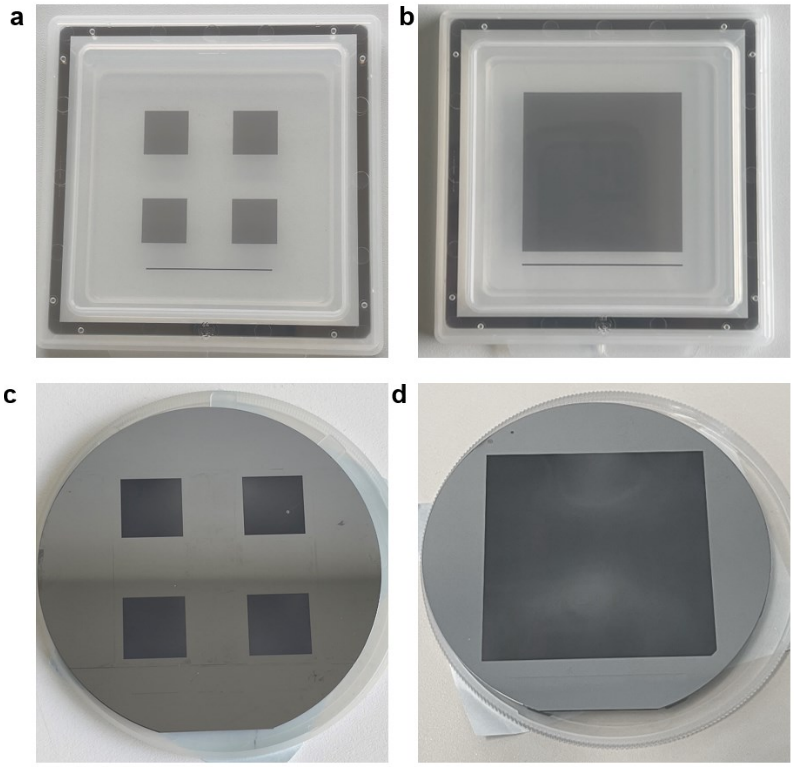

Supplementary Fig. 3. Photomasks and silicon moulds for PMMM fabrication. **a**, The 5-inch chrome photomask for small-scale (3.2 cm^2) PMMM fabrication. **b**, The 5-inch chrome photomask for larger-scale (42.2 cm^2) PMMM fabrication. **c**, The silicon mould is patterned with 1 million inverted micro-pyramids per square centimetre. Each patterned block is 3.2 cm^2. **d**, The larger-scale silicon mould with an area of 42.2 cm^2.

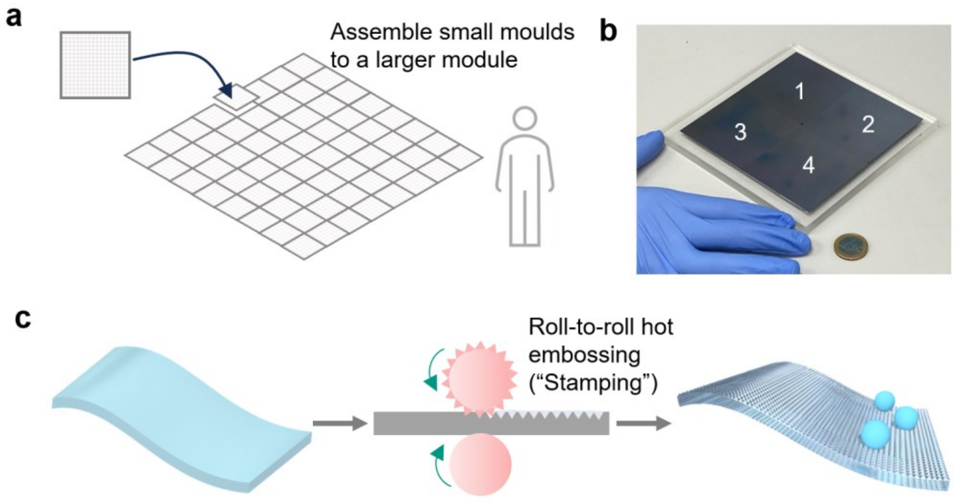

Supplementary Fig. 4. Upscaling strategy. **a**, Straightforward assembly of a matrix of small silicon wafer moulds. This technique allows for the creation of a larger-sized module suitable for the production of extensive PMMM films. **b**, An example where four small silicon pieces (6.5 cm × 6.5 cm) are combined to form a larger module (13 cm × 13 cm), with a 1 Euro coin provided for scale reference. **c**, Hot-embossing technology for the fabrication of large-scale PMMM films. Hot embossing is renowned for its ability to consistently produce high-quality microstructures across expansive areas. This technique's precision and scalability make it an ideal choice for producing PMMM films on a larger scale.

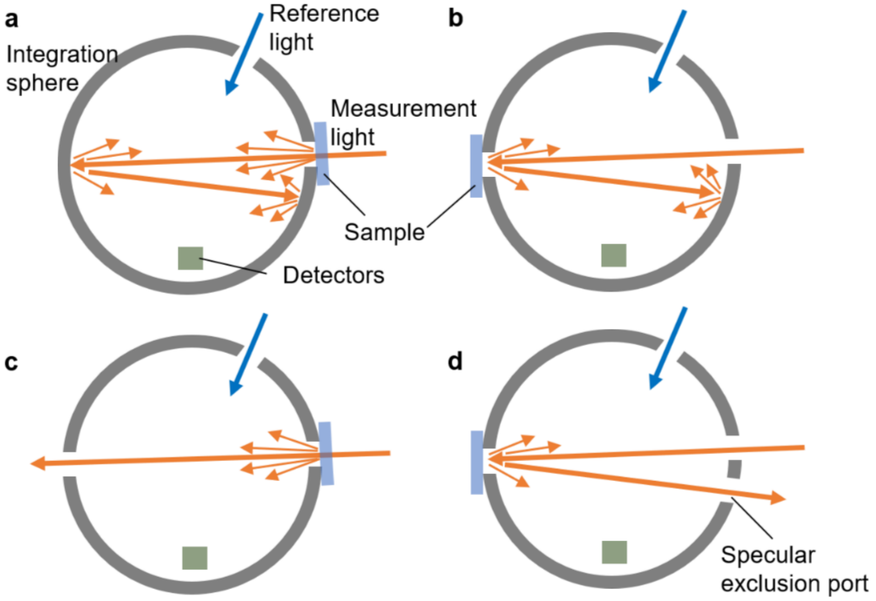

Supplementary Fig. 5. **Illumination and light collection geometries (top view). a,** global transmittance. **b,** global reflectance. **c**, diffuse transmittance. **d**, diffuse reflectance. In these setups, either direct or diffuse light is captured using an integration sphere, which features a highly reflective internal surface. This light is then directed to detectors located at the bottom of the sphere for accurate measurement. Prior to conducting these measurements, the spectrophotometer is meticulously calibrated to ensure precision and reliability in our results.

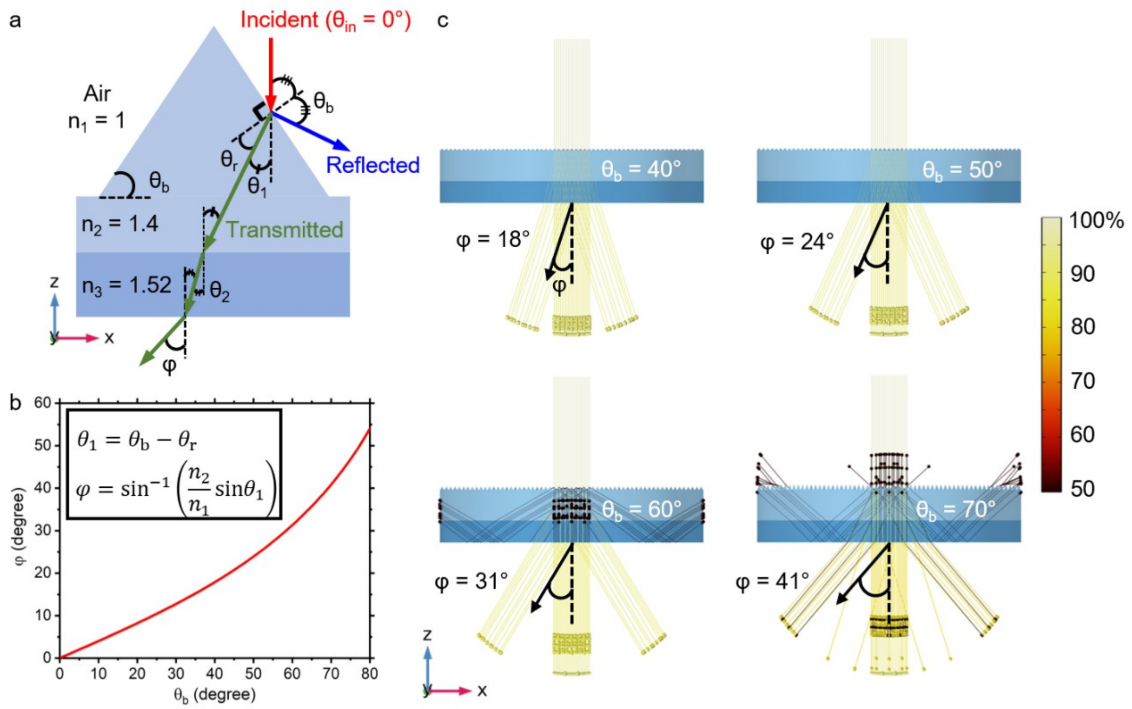

Supplementary Fig. 6. Correlation between the diffusion angle and the base angle of the micro-pyramid. a, Schematic illustration of the correlation between the diffusion angle and the base angle of the micro-pyramid for $\theta_{in} = 0°$. θ_b is the micro-pyramid's base angle. The incident angle at the micro-pyramid's surface equals θ_b. θ_r is the refraction angle. **b**, θ_b dependency of φ. The plot originates from the equations presented in the figure. Those equations are derived based on Snell's law and the schematic illustration. The plot suggests that φ increases with an increase in θ_b. **c**, Side views of ray-tracing maps of the PMMM with $\theta_b = 40°$, 50°, 60°, and 70°. The maps depict that sunlight can be distributed in a wider angular range by increasing θ_b.

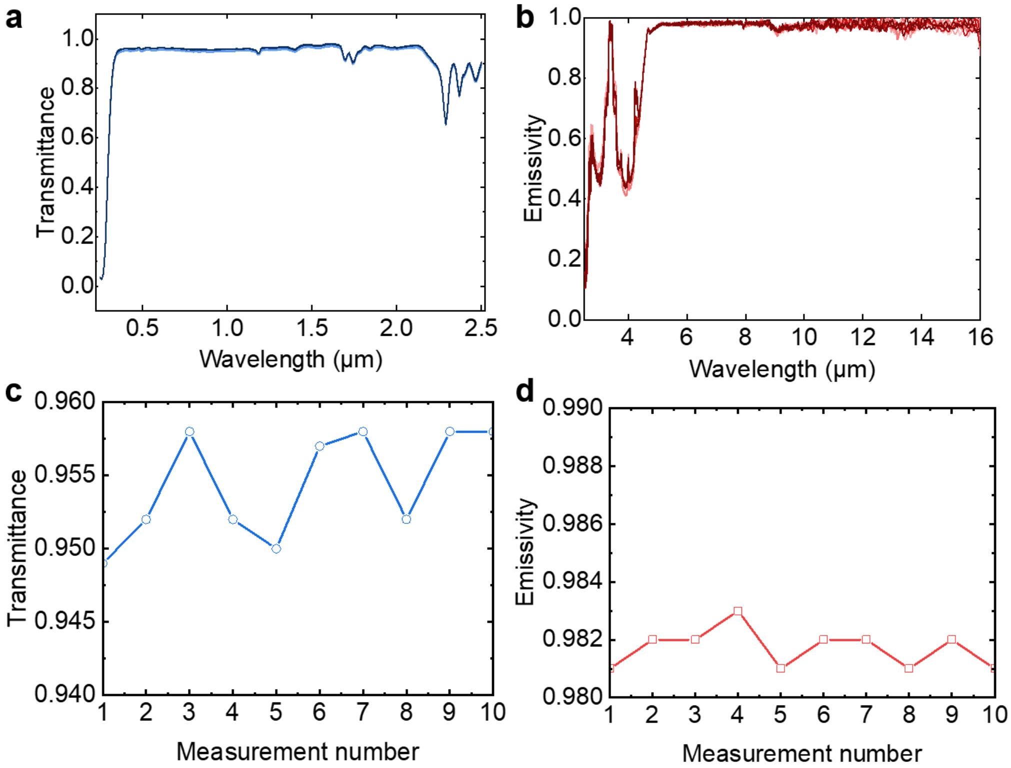

Supplementary Fig. 7. Error of 10-times repeated measurements. a, Variations in transmittance observed across 10 measurements. We would like to clarify that the Agilent Cary 7000 spectrophotometer, which we utilized for our measurements, has a very low inherent measurement error. Despite this high accuracy, we recognize that the primary source of uncertainty in our experiments could not come from the instrument itself but rather from the samples positioning within it. This uncertainty arises because even minor variations in the placement of the samples can affect the path and angle of light as it interacts with the sample, leading to slight variations in the measurement results. Small deviations in the angle or position can change the way light is scattered, reflected, or absorbed, hence impacting the spectrophotometric readings. To address this concern, we have re-evaluated the optical properties and emissivity of our samples. This involved conducting 10 separate measurements, removing and then remounting the sample in the device each time. **b,** Variations in emissivity observed across 10 measurements. **c**, Average transmittance values in the 0.3–2.5 μm range from these 10 measurements. The mean value is 0.954±0.004. **d**, Average emissivity values in the 8–13 μm range from these 10 measurements. The observed uncertainty is as low as 0.2%. The mean value is 0.9817±0.0006. (**Uncertainty analysis**: The measurements of the reflection and transmission properties were carried out using UV-Vis-NIR Spectrophotometer (Agilent Cary 7000) across a wide range of wavelengths (300–2500 nm). The instrument was equipped with an integrated sphere with a highly reflective inner surface to collect both diffused and direct light. All measurements were done using a NIST calibrated diffuse reflectance standard. The emissivity was measured by a Fourier-transform infrared spectroscopy (Bruker Vertex 70) which was equipped with an integrated sphere (A562) with a highly infrared reflective inner surface coated with gold. The equations used to calculate the values of τ_{g_ave}, τ_{dif_ave}, τ_{vis_ave} and ε_{ave} can be found in the Method section. Corresponding uncertainties for values above were calculated according to a published guide[2]. Random errors impact was estimated using acquired transmission/reflection spectra and 10-times repetitive

measurements with a repositioned sample, whereas the systematic uncertainties (type B) were valued with best knowledge using a technical specification of both Cary 7000 (DRA attachment) and Bruker Vertex 70. All systematic uncertainties were quadratically summed and after added to random errors to get a total uncertainty of the measurement. For the measurements in UV/Vis/NIR spectra, whereas the random error and all systematic uncertainties caused by nonideality of the system are quite low (0.1–2 rel.%), depending on an acquired value (for ~100% and 10% transmission/reflection, accordingly). The total uncertainty is mainly governed by a uncertainty of reflectivity estimation of the calibrated standard (~0.5% in UV/Vis/NIR ranges 0.3–2 μm and ~3% in the NIR range (>2 μm)) or an estimated bias of the uncalibrated reference golden port in FTIR measurements. One should however point out, that as the average transmission in our calculation is weighed by the AM1.5 solar spectrum, main intensity of which is concentrated in the visible range, a moderately high uncertainty of the standard in NIR range has only a minor impact on the resulting total uncertainty. In FTIR range, both random noise and estimated systematic uncertainties are relatively higher and are about 2–5 rel.%. It is worth to note, that in a mutual comparison between glass and PMMM systems, the main uncertainty, caused by a reflectivity of the reference port close to 100% but having unknown absolute value, is equally applied to glass and PMMM samples. Summary of calculated integrals with uncertainties: (1) Glass sample: τ_{g_ave} = 0.906±0.004; τ_{dif_ave} = 0.014±0.0003; τ_{vis_ave} = 0.909±0.004; ε_{ave} = 0.87±0.05; (2) PMMM sample: τ_{g_ave} = 0.949±0.004; τ_{dif_ave} = 0.731±0.003; τ_{vis_ave} = 0.952±0.025; ε_{ave} = 0.98±0.05. Uncertainties A and B type is summarized in **Supplementary Table 1** at the end of this supplementary file).

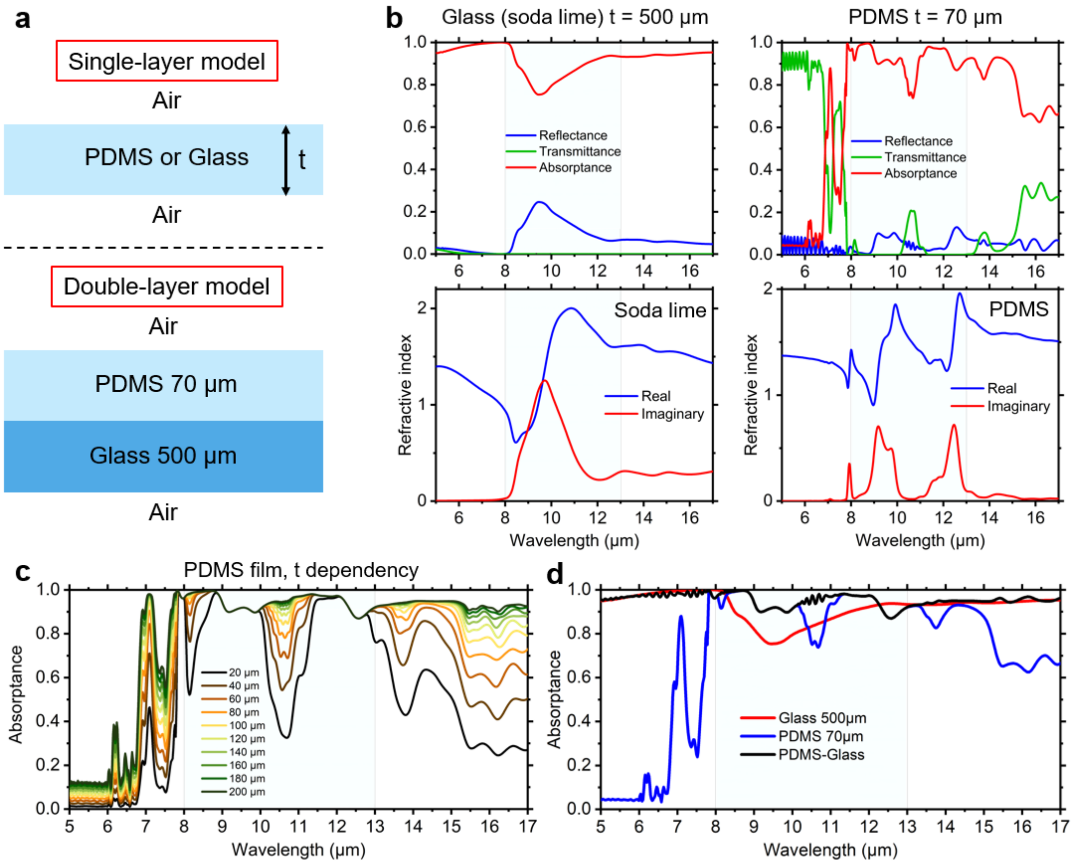

Supplementary Fig. 8. Modelling results of optical properties of flat films in the mid-infrared range via transfer matrix method (TMM). **a**, Schematic illustrations of the single-layer and double layer models for the TMM calculations. **b**, Optical properties of a soda-lime glass and a polydimethylsiloxane (PDMS) film. Top row: reflectance, transmittance, and absorptance of the glass with thickness t = 500 μm and the PDMS film with t = 70 μm. The spectra are calculated using the single-layer model. Bottom row: the blue and red lines are for the real and imaginary parts of the refractive indices, respectively. For the glass, it possesses strong absorption in the mid-infrared (MIR) range, but its absorption is weakened in the atmospheric transparency window (ATW, 8 – 13 μm) because of its reflection. In contrast, the PDMS film's absorption is strong especially in the ATW. The PDMS film, however, cannot achieve 100% absorptance in the ATW since the PDMS film also reflect an incoming radiation. The reflection properties of the glass and PDMS film can be understood from their refractive indices. The imaginary part of a refractive index, which is called extinction coefficient, is directly related to the absorption properties of a material. Soda-lime glass and PDMS has large extinction coefficient in the ATW; therefore, one might expect that they can be an ideal material for radiative cooling. However, the real parts of their refractive indices dynamically changes at around the peaks of the extinction coefficients. This causes large refractive index mismatch at the interface between air and the films, resulting in strong reflection. **c**, Thickness dependency of the absorption properties of the PDMS film. The absorption is calculated using the single-layer model with varying thickness in a range of 20–200 μm. The absorption properties of a flat film are governed by Beer's law, which states that the film possess strong absorption if its extinction coefficient is large, and the film's absorption becomes strong with an increase in thickness. For the PDMS film, its extinction coefficient is large at around 9.5 μm and 12.5 μm. Thus, it can possess strong absorption at around those wavelengths even though t is small. Based on Beer's law, the PDMS film's absorption is expected to become even stronger with increasing thickness. This is not true for PDMS at around 9.5 μm and 12.5 μm, since the reflection at the air-PDMS interface limits the

absorption capability of the PDMS film. In wavelength regions where the dispersion of the refractive index is weak, the absorption of the PDMS film can be improved just by increasing thickness because the incoming radiation is mostly transmitted at the air-PDMS interface. **d**, Absorption spectra of the PDMS film with t = 70 μm (blue line). By combining those materials, nearly blackbody-like absorber can be realized in the MIR. But there is still room for improvement in the ATW. In order to improve the PDMS-glass film's absorption, the surface the PDMS film needs to be textured by a microstructure.

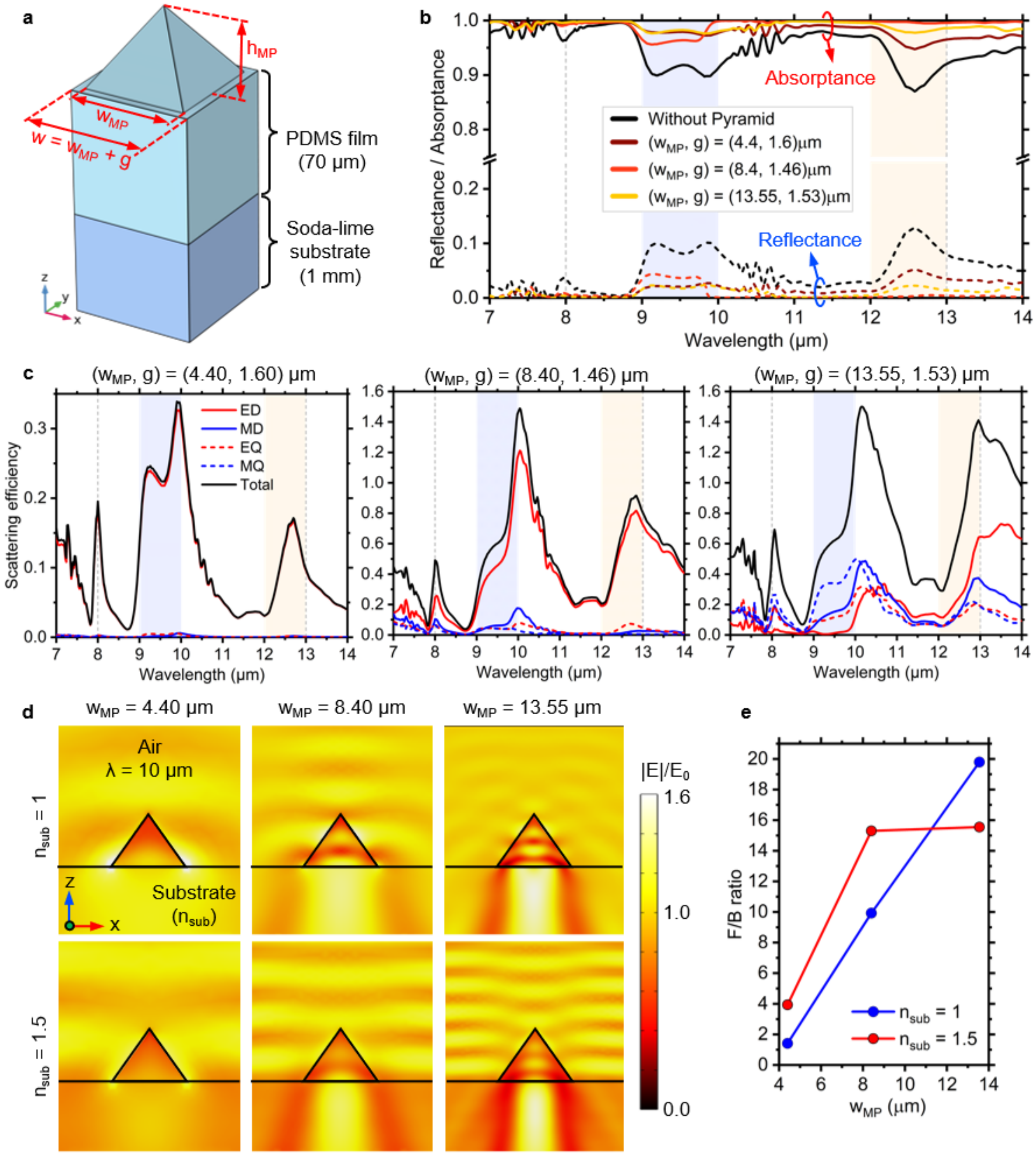

Supplementary Fig. 9. Absorption enhancement mechanism of the PMMM in the MIR range. a, Simulation model of the PMMM. Wave-optics simulations were conducted using COMSOL Multiphysics (the finite element method). The model comprises a PDMS micro-pyramid, PDMS film, and soda-lime substrate. The top micro-pyramid has the width and height of w_{MP} and h_{MP}, respectively. The height is given by $h_{MP} = (w_{MP}/2)\times\tan(54.7°)$, where 54.7° is the base angle of the fabricated micro-pyramid. To reduce computational cost to simulate the thick film and substrate, the transition boundary conditions were applied on their bottom surfaces. The perfectly matched layers (PMLs) were set on top and bottom of the calculation domain to consider infinitely large air space. The simulation model was used as a unit cell, and the periodic boundary conditions were applied in the *x*- and *y*- directions. The periodicity of the unit cell was given as $w = w_{MP} + g$, where g is the gap between the micro-pyramids. In

the simulation, the x-polarized incident wave propagated along the $-z$ direction. **b**, Comparison of different micro-pyramids' optical responses in the atmospheric window range of 8–13 μm. The solid and dashed lines are for absorptance and reflectance, respectively. The reflectance and transmittance of the simulation model were calculated, and absorptance was obtained by 1-(reflectance)-(transmittance). The micro-pyramid structures labeled as w = 6 μm, 10 μm, and 16 μm in Fig. 3b of the main text were simulated. In the simulation, the parameter sets of (w_{MP}, g) = (4.4, 1.6) μm, (8.40, 1.46) μm, and (13.55, 1.53) μm were used for those micro-pyramids. The parameter sets were obtained from the SEM images. The optical response of a flat surface (without micro-pyramid) was also simulated. Compared with the flat surface and the textured surfaces, it is found that the textured surfaces have higher absorptance, especially around the wavelength ranges of 9–10 μm and 12–13 μm (blue and yellow shaded areas). The absorption is enhanced because the reflection is weakened at the air-PDMS interface by the micro-pyramid structure. **c**, Resonance mode analysis. The resonance modes of the micro-pyramids were extracted by using the simulation model illustrated in (a) and employing Cartesian multipole decomposition (CMD)[3]. In general, CMD is used for single particles[4], but it can also be applied to periodic systems[5]. From CMD, four resonance modes were obtained: electric dipole (ED), magnetic dipole (MD), electric quadrupole (EQ), and magnetic quadrupole (MQ) moments. In the plot, those resonance modes are characterized as scattering efficiencies. The total scattering spectrum of the micro-pyramids is given by the sum of the scattering spectra of those modes. For w_{MP} = 4.4 μm, the total scattering is dominated by the ED resonance. With increasing w_{MP}, the higher-order modes contribute to the total scattering. **d**, Electric field distribution of the single micro-pyramid placed on a substrate with a refractive index of n_{sub}. The maps present the absolute value of the electric field (E) normalized by the electric field of the incident wave (E_0) at the wavelength of 10 μm. It must be mentioned that a simulation model different from the one shown in (a) was used to simulate the single micro-pyramid. For this simulation, the periodic boundary conditions were not applied, but the PMLs were set around the micro-pyramid. For n_{sub} = 1 (air), the micro-pyramid with w_{MP} = 4.4 μm shows the dipolar-like electric field distribution, where the strong electric near fields are created around the bottom apexes. When n_{sub} increases to 1.5, the near fields weaken since the created electric fields couple into the substrate, resulting in the incident light being strongly scattered into the substrate[6–9]. For the larger micro-pyramids (w_{MP} = 8.40 and 13.55 μm) with n_{sub} = 1, their near-field distributions are more complex compared with the smallest micro-pyramid because of their higher-order modes. In a similar manner to the smallest micro-pyramid, the near fields of the larger micro-pyramids become weak when n_{sub} = 1.5. **e**, Forward to backward scattering (F/B) ratio. The F/B ratio was calculated using the same simulation model for (d) to understand the scattering properties of the single micro-pyramid quantitatively. The forward and backward scatterings were defined by the powers of the scattered waves below and above the substrate, respectively. The F/B ratio is plotted as a function of w_{MP}. The blue and red marks are for n_{sub} = 1 and 1.5, respectively. For w_{MP} = 4.4 μm and n_{sub} = 1, the F/B ratio is about 1.4, meaning that the smallest micro-pyramid scatters the incident wave almost symmetrically into the forward and backward directions because of its ED resonance[10,11]. When the smallest micro-pyramid is located on the substrate with n_{sub} = 1.5, the F/B ratio is increased to about 4, which proves that the forward scattering becomes strong for a high n_{sub}[6–9]. For w_{MP} = 8.4 μm, similarly to the case of w_{MP} = 4.4 μm, the F/B ratio can be significantly improved by increasing n_{sub}. When w_{MP} is increased to 13.55 μm, the F/B ratio is high regardless of

whether there is a substrate on the micro-pyramid′s bottom, since the largest micro-pyramid possesses strong forward scattering by its complex resonance state[11–13].

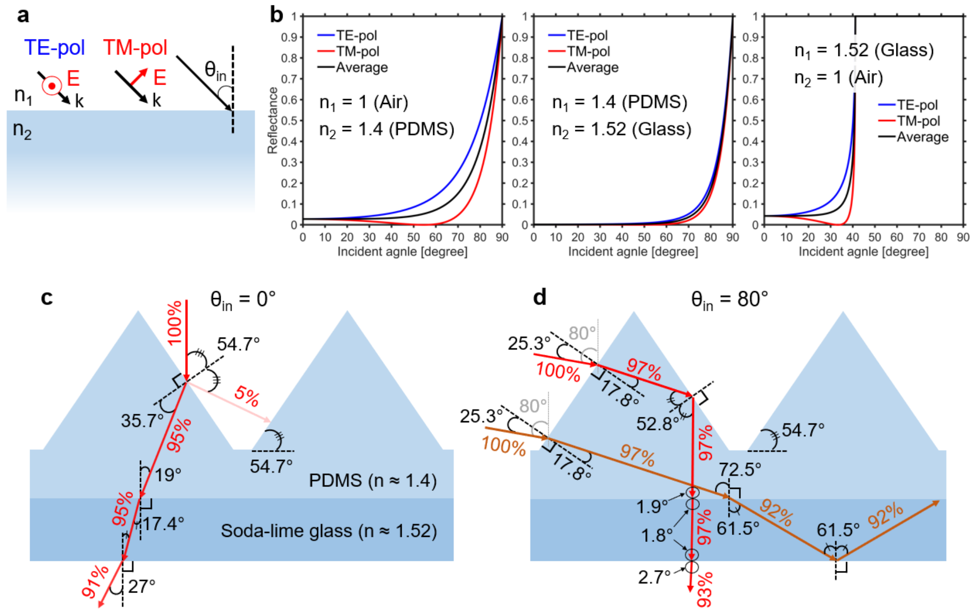

Supplementary Fig. 10. Optical properties of the micro-pyramid structure in the visible range. a, Schematic illustration of an incident light at an interface between two media with different refractive indices $n1$ and $n2$. θ_{in} is the incident angle. For this calculation, it is considered that an incident wave with transverse-electric (TE) or transverse-magnetic (TM) polarizations strikes the interface at an incident angle of θin. Also, it is considered that PDMS and glass have refractive index of 1.4 and 1.52, respectively. **b**, Reflection properties of different interfaces. Reflectance is plotted as a function of the incident angle. For the air-PDMS interface, corresponding to the micro-pyramid's surface, the reflectance becomes zero at Brewster's angle ~54.5° for TM-polarization. The reflection at the PDMS-glass interface is very weak because of the small refractive index contrast. The critical angle of the glass-air interface is $\theta_{in} \approx 41°$. Thus, when the incident light passes through the structure and strikes the bottom glass-air interface at $\theta_{in} > 41°$, the light is reflected by the total internal reflection. Since we are interested in that the incident light is sunlight, which is unpolarized, we also plotted reflectance for an unpolarized light by taking the average of reflectance for TE- and TM-polarizations (black lines). **c**, Schematic illustration of the light distribution properties of the micro-pyramid structure for normal incidence. In the main text, we show that when the incident angle is 0°, the incident light is distributed into five directions. This phenomenon can be described using Snell's law. When the reflection of a planer film is considered, it can be imagined that a reflected ray goes in the direction of the light source; therefore, the reflected ray is measured as reflectance. In contrast, the ray reflected at the micro-pyramid's surface goes back to the PMMM because of the inclined surface of the micro-pyramid. This reflected ray is counted as transmittance. Thus, the PMMM can possess a higher transmittance compared with the planar glass substrate. **d**, Schematic illustration of the light distribution properties of the micro-pyramid structure when $\theta_{in} = 80°$. This light distribution phenomenon for the oblique incidence is attributed to the fact that the total internal reflection occurs inside the pyramid structure. In order to fully understand the optical properties of the pyramid structure for $\theta_{in} = 80°$, two optical paths must be considered. One is that the incident light interacts with the pyramid structure twice, and the total internal reflection occurs at the second interaction (the critical angle at PDMS-air interface ~45.6°), which contributes to the light distribution to the bottom of the structure (red arrows). Another is that the incident light is refracted at

the air-PDMS interface and strikes the bottom glass-air interface at the incident angle larger than the critical angle of 41° (orange arrows). This light is reflected back to the top side of the structure.

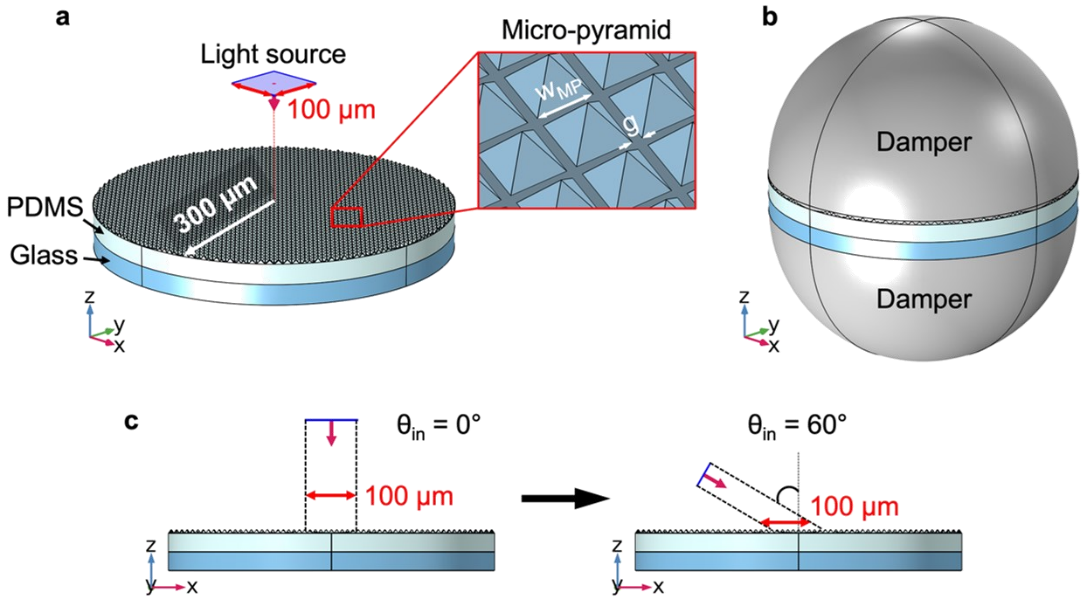

Supplementary Fig. 11. Ray-tracing simulation model description. a, Simulation model for the ray-tracing simulation. The model considers that the PDMS micro-pyramid with the width (w_{MP}) and the gap (g) are periodically placed on the PDMS-glass substrate, whose radius is 300 μm. In this study, w_{MP} = 8.4 μm and g = 1.46 μm were used. The periodicity of the micro-pyramid is given as $w = w_{MP} + g$. Those values were determined based on the SEM images of the micro-pyramid labeled as w = 10 μm in Fig. 3b of the main text. The pyramids are oriented so that their surfaces face to the x- and y-directions. The thicknesses of the PDMS film and glass substrate are 70 μm and 50 μm, respectively. In the experiment, the thickness of the glass substrate is 1 mm. Compared with this value, the glass substrate of the simulation model is thin. This is to prevent the rays from traveling for a long distance inside the glass substrate and striking the glass substrate's sidewall before exiting from its bottom. The light distribution properties of the PMMM are attributed to light reflections and refractions. Those optical phenomena are independent of the glass substrate's thickness; therefore, the simulation model can describe the light distribution properties of the PMMM well even though the thin glass substrate is used. The refractive indices of PDMS and glass are 1.40 and 1.52, respectively. The size of the light source is 100 μm × 100 μm for normal incidence. The source emits 500 rays. In this simulation, reflected and refracted rays with an intensity lower than 5% of the incident rays are omitted. **b**, A boundary condition to strop the propagations of the rays is applied around the PMMM. **c**, Side views of the simulation model for different incident angles. For oblique incident angles, the size of the light source changes so that the incident rays strike the PMMM without changing the incident area of 100 μm × 100 μm.

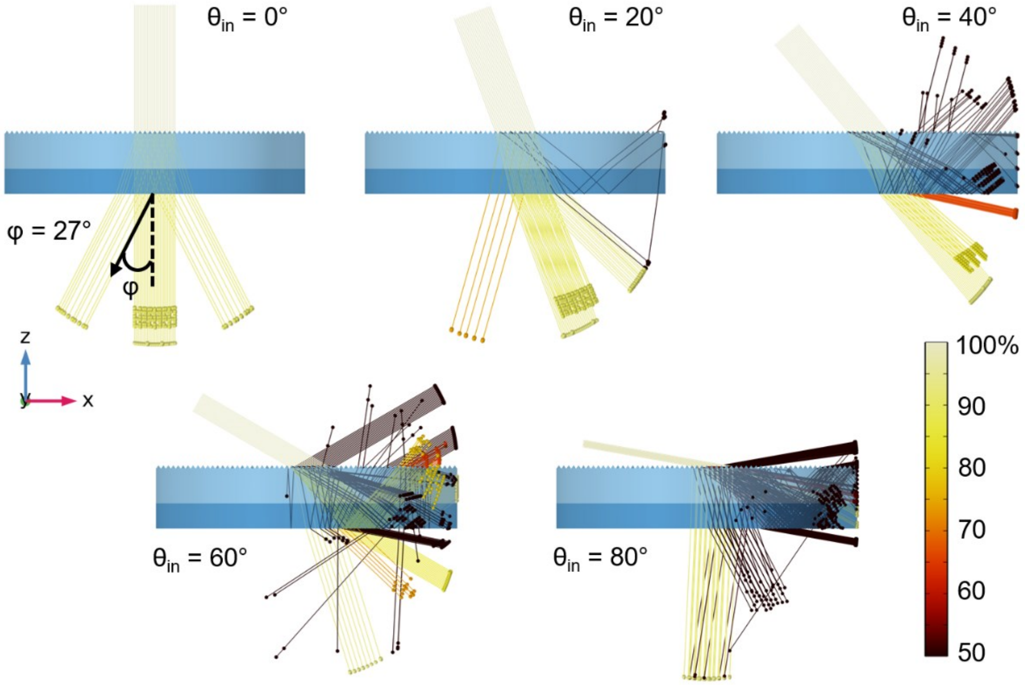

Supplementary Fig. 12. Side views of the ray propagation maps. The ray-tracing maps of Fig. 3a of the main text are presented from their side for a better understanding of the light distribution properties of the PMMM. In addition to θ_{in} = 0°, 40° and 80°, θ_{in} = 20° and 60° are also shown. For θ_{in} = 0°, φ is measured using the map, resulting in φ = 27°.

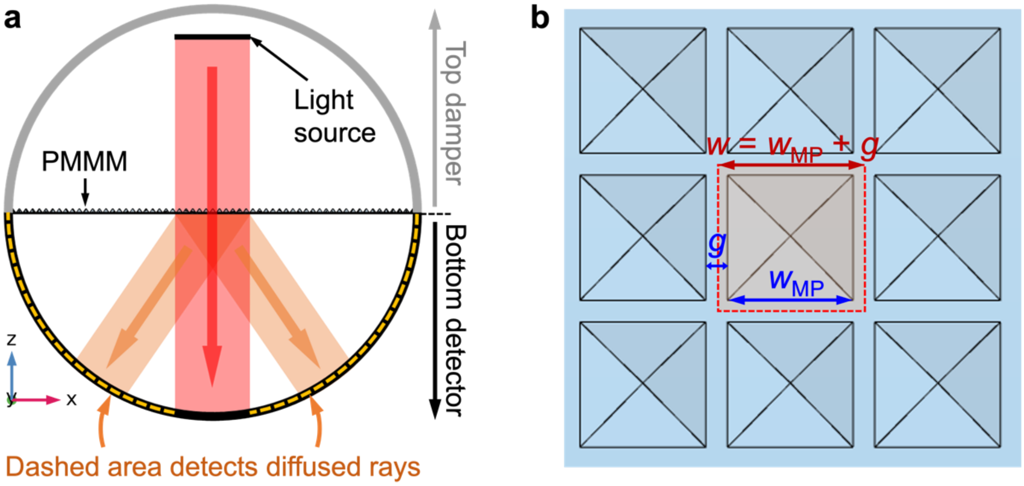

Supplementary Fig. 13. Global and diffused transmittance. a, Schematic illustration of the side view of the simulation model used to calculate τ_{g_ave} and τ_{dif_ave}. The light source emits 10000 rays so that the incident rays can uniformly strike the PMMM. In order to calculate τ_{g_ave} and τ_{dif_ave} accurately, the simulation considers the reflected and refracted rays with an intensity down to 0.1% of the incident rays' intensity. The top and bottom of the calculation domain are enclosed by a damper and a power detector, respectively. The thicknesses of the PDMS film and glass substrate are decreased to 1 μm so that any rays do not strike the sidewall of the PMMM, and all rays can reach either the top damper or bottom detector. τ_g is measured by integrating the powers of the transmitted rays over the bottom detector. τ_{dif} is calculated by integrating the powers of the transmitted rays that do not pass through the PMMM straight. The simulation result gives $\tau_{g\_ave\_sim} = 93.3\%$ and $\tau_{dif\_ave\_sim} = 72.2\%$, which are close to the experimental results of $\tau_{g\_ave\_exp} = 95\%\pm1\%$ and $\tau_{dif\_ave\_exp} = 73\%\pm1\%$. **b**, Schematic description of the correlation between τ_{dif} and the microstructure of the PMMM. The red shaded area indicates the unit cell of the microstructure. The width of the micro-pyramid is given as w_{MP}, and g is the gap between the micro-pyramids. All rays that pass through the micro-pyramid's surface are refracted or reflected, resulting in the diffused rays. On the other hand, the rays striking the flat gap space do not become the diffused rays. Therefore, τ_{dif} may be estimated by the ratio of the area of the micro-pyramid structure to the area of the unit cell. By using $w_{MP} = 8.4$ μm and $g = 1.46$ μm, τ_{dif} is estimated as $w_{MP}^2/w^2 = 0.726$, which agrees with the simulation result.

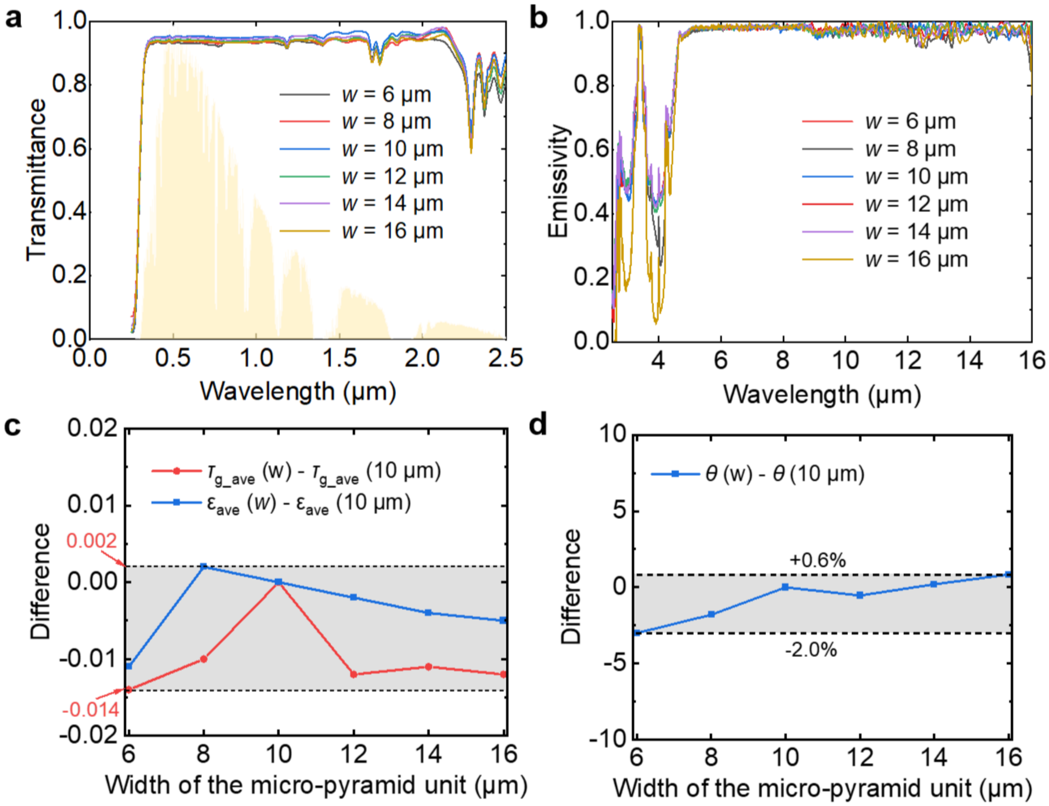

Supplementary Fig. 14. **Performance of PMMM samples with various micro-pyramid unit sizes. a**, Global transmittance of the PMMM samples. **b**, Emissivity of the PMMM samples. The variations in both spectrally-weighted average transmittance (τ_{g_ave}) and average emissivity (ε_{ave}) across pyramid sizes ranging from 6 to 16 μm are relatively minor. **c**, The differences in τ_{g_ave} and ε_{ave} for pyramid units of different sizes against the 10-μm-wide pyramid units. The observed variations in τ_{g_ave} and ε_{ave} are less than 0.014 across this size range. **d**, The differences in contact angle θ for pyramid units of different sizes against the 10-μm-wide units. The variation in contact angle is less than 3° across the range of pyramid sizes, translating to a change of less than 2%.

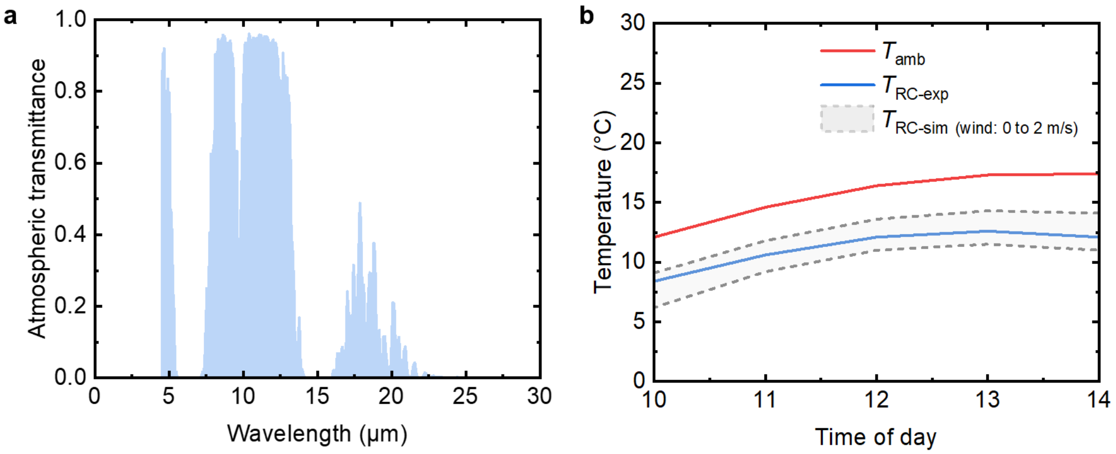

Supplementary Fig. 15. **Atmosphere transmittance and model validation. a**, Atmospheric transmittance for the modelling[14]. Of note is that the modelling method refers to the recently corrected model by Raman's group, as detailed in Method section[15]. **b**, Simulation results compared to experimental results (The experiment was conducted by Raman *et al.* in Stanford, California, in mid-December 2013). To ensure the accuracy of our revised model, we conducted a comparison against the experimental results from Raman et al.[14] This comparison demonstrates a good alignment between our simulation outcomes and the referenced experimental data. The heat convection coefficient was calculated by $h_{\mathrm{wind}} = 2.5 + 2*v_{\mathrm{wind}}$, where V_{wind} is the wind speed (Zhao et al.[16]).

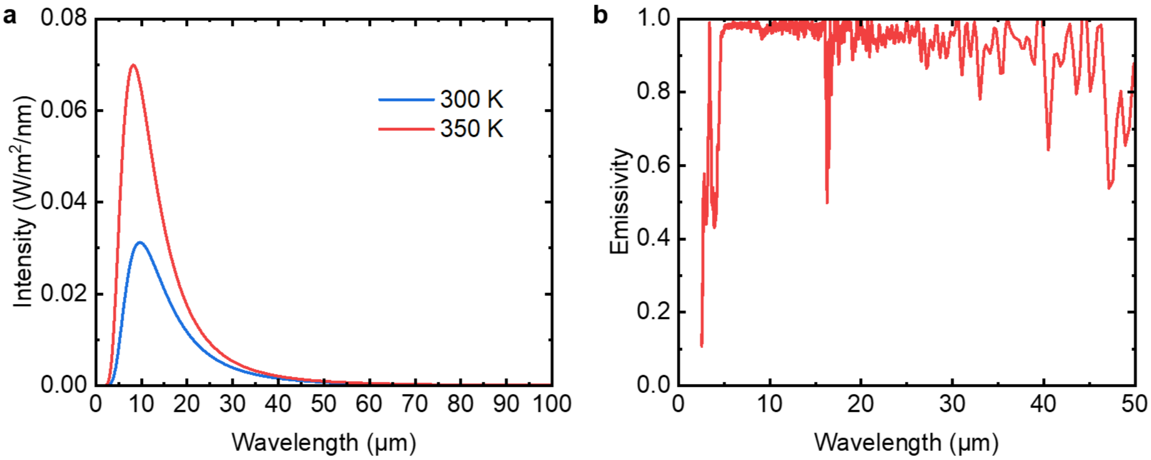

Supplementary Fig. 16. **Thermal radiation models. a**, Thermal radiation spectrum of black body. The thermal emission from a blackbody at temperatures of 300 K and 350 K predominantly occurs within the 3–50 μm wavelength range. **b**, Emissivity of PMMM over the 3–50 μm wavelength range.

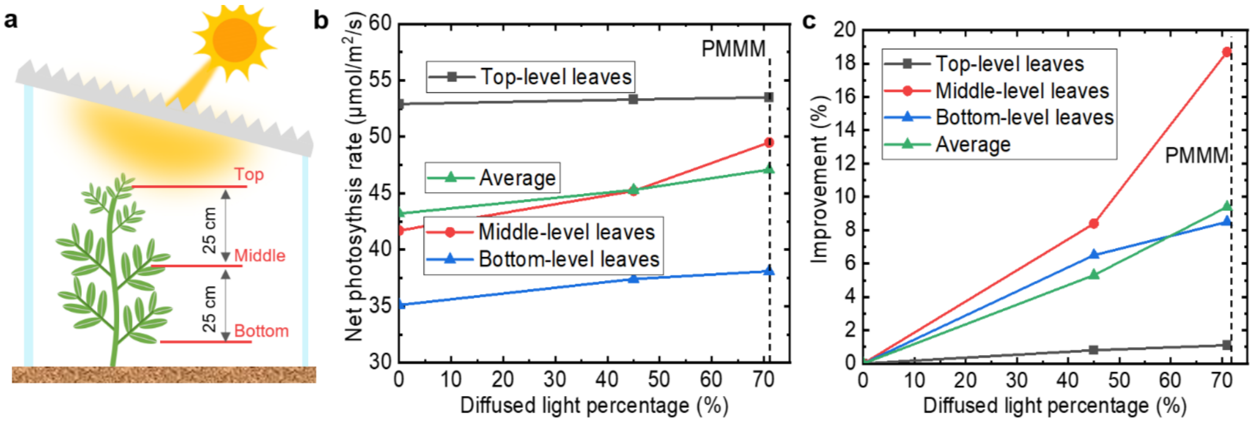

Supplementary Fig. 17. Impact of diffused light on the photosynthesis rate. a, Schematic of the testing tomato plants in a greenhouse with light diffusor. The photosynthesis rates at the top, middle and bottom levels of the tomato plants were monitored by Li et al.[17]. **b**, The impact of diffused light on the photosynthesis rates of the top-, middle- and bottom-level leaves. The diffused light can significantly improve the photosynthesis rates of middle-level leaves. The average photosynthesis rates were 41.7 and 49.5 μmol/m^2/s when the diffused light percentages were 0% and 71%. **c,** The relative improvement of photosynthesis rates for different diffused light percentages. The PMMM film (diffused light percentage τ_{dif} = 72%) is estimated to be able to improve the photosynthesis rate by 9.5% according to the experimental data[17].

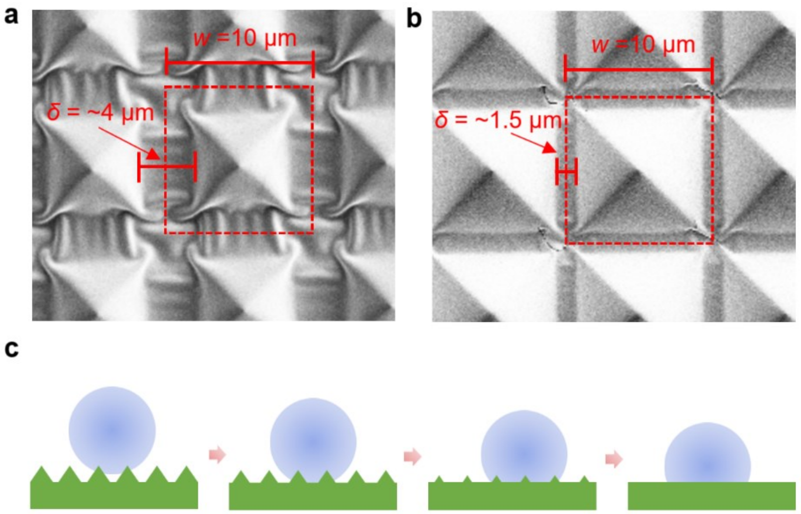

Supplementary Fig. 18. SEM images of PMMM samples with different gaps and the schematic of wettability change. **a**, SEM image of PMMM sample with δ = ~4 μm gap between adjacent pyramids. **b**, SEM image of PMMM sample with δ = ~1.5 μm gap. **c**, Schematic illustrating the inverse relationship between δ and the contact angle, with a flat surface ($\delta \approx 10$ μm) as the extreme condition. The figure illustrates that an increase in the spacing (δ) between the micro-pyramids leads to a corresponding increase in the flat area of the PMMM. This larger flat area facilitates the spreading of water droplets on the PMMM surface, thereby reducing the contact angle. For example, the PMMM sample with δ = ~1.5 μm exhibits a higher contact angle of 152° compared to the 128° of the sample with δ = ~4 μm. This behavior aligns with the principles of the *Wenzel* equation: $\cos\theta = r{\cdot}\cos\theta_0$, where θ represents the contact angle on the structured surface, θ_0 is the contact angle on the flat base material, and r is the roughness ratio (the ratio of actual to projected solid surface area). As δ increases, the roughness ratio r decreases, leading to a reduction in the contact angle θ.

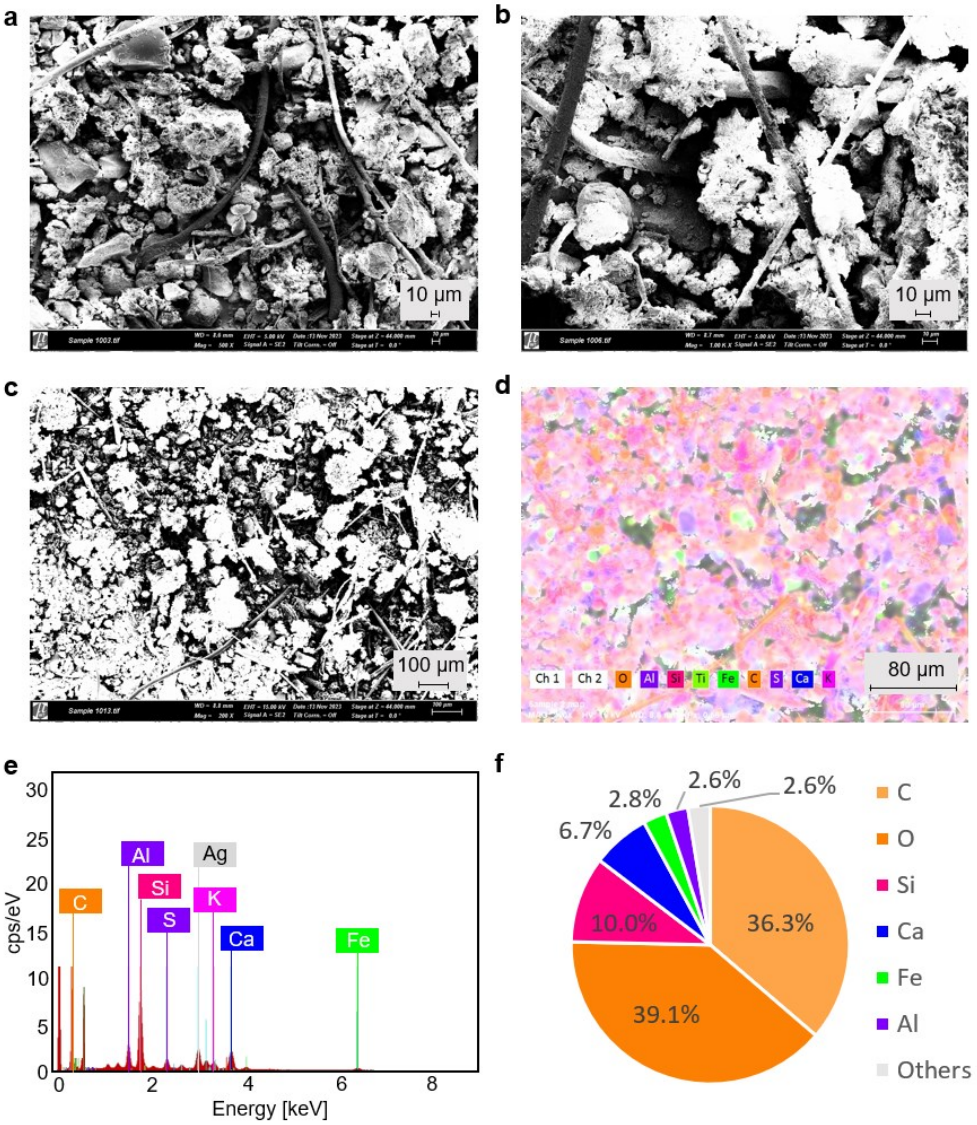

Supplementary Fig. 19. SEM/EDX characterization of the dust. a, SEM image of the dust particles (selected part 1). **b**, SEM image of the dust particles (selected part 2). **c**, SEM image of the dust particles (selected part 3). the dust particle sizes predominantly larger than 10 μm, with a majority being silicate and carbonate minerals, which are common constituents of atmospheric dust. **d**, Element distribution of the dust particles. A majority are silicate and carbonate minerals, which are common constituents of atmospheric dust. **e**, Elements of the dust particles. **f**, Element percentages of the dust particles. The anti-dusting capabilities are the result of a synergy between the surface microstructure and the inherent material properties. The PMMM film is textured with micro-pyramid features that replicate the hierarchical micro-and nano-scale roughness found in superhydrophobic natural surfaces, like lotus leaves. This structure reduces the contact area between dust particles and the surface, significantly lowering the adhesion force due to the Cassie-Baxter wetting state, where air pockets are trapped underneath water droplets. The polydimethylsiloxane used in the PMMM provides a low surface energy, which inherently repels water and thus, aqueous-based dirt and dust particles. This hydrophobic nature is further enhanced by the micro-pyramid surface patterning, contributing to a superhydrophobic surface

with a high contact angle for water droplets. When water droplets impact or roll across the PMMM surface, they collect dust particles through adhesion and momentum transfer. The water droplets, with the entrapped dust, then readily roll off due to the low adhesion, effectively cleaning the surface. This process is akin to the "lotus effect," where rainwater washes away contaminants.

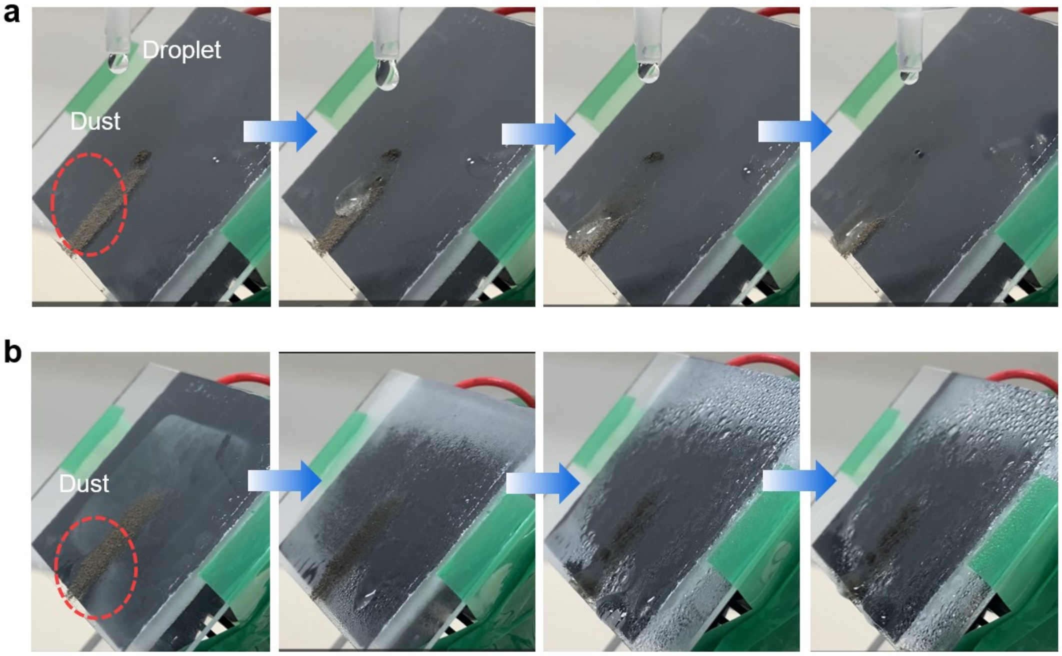

Supplementary Fig. 20. Cleaning processes of a glass plate. a, Active cleaning process by water droplets (indoor lab simulated experiment). The glass surface, being hydrophilic, struggles with active cleaning by water droplets. We used the same amount of water droplets to clean the glass surface as we used to clean the PMMM samples. The droplets tend to adhere to the glass, making it difficult to effectively dislodge dust with the limited amount of water droplets. More water is required to remove the dust further and fully on the glass surface. **b**, passive cleaning process by dew (indoor lab simulated experiment). The hydrophilic nature of the glass surface leads to the formation of a dew film instead of discrete droplets, lacking the roll-off effect necessary for effective cleaning. As a result, the wetted dust remains adhered to the glass surface, making it more challenging to remove effectively at the end of the dew formation process. In contrast, the hydrophobic PMMM surface easily forms noticeable dew droplets that can roll off, sweeping across the surface and efficiently removing dust.

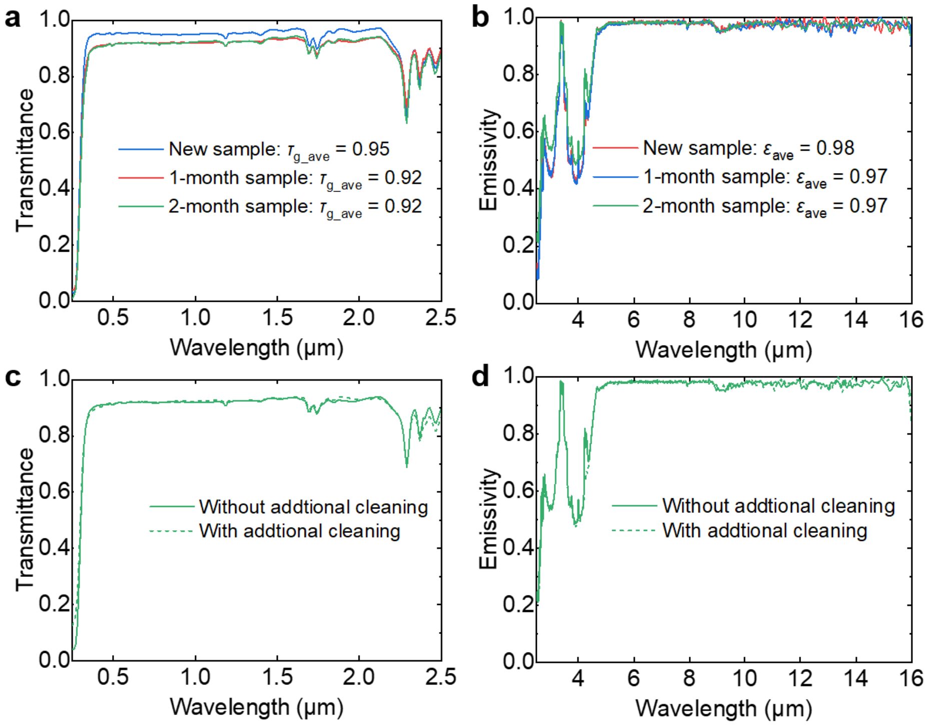

Supplementary Fig. 21. **Degradation of PMMM in outdoor testing. a**, Transmittance reduction of PMMM samples after 1-month and 2-month outdoor exposure. To provide a more comprehensive assessment of our metamaterial's stability, we conducted a real-world test by exposing samples to the outdoor environment in Karlsruhe, Germany, for durations of one and two months. Specifically, the 1-month sample was exposed from 14th November 2023 to 15th December 2023, while the 2-month sample was left outdoors from 14th November 2023 to 15th January 2024. The results show a transmittance reduction of 3% after one month. It's important to note that the transmittance underwent only marginal changes with the extension of exposure time to two months. This data suggests a relative stability of the PDMS-based metamaterial under extended outdoor environmental conditions. The performance of both the 1-month and 2-month samples was assessed directly post-exposure, without any cleaning, upon their return from the outdoor environment. Therefore, the observed changes in performance can be attributed to a combination of aging and soiling effects. **b**, Emissivity reduction of PMMM samples after 1-month and 2-month outdoor exposure. The results show an emissivity reduction of only 1% after 1-month and 2-month outdoor exposure. **c**, Transmittance of the 2-month sample without and with additional cleaning. **d**, Emissivity of the 2-month sample without and with additional cleaning. To disentangle the impacts of aging from soiling, the 2-month sample underwent a cleaning process using distilled water. We then reassessed its transmittance and emissivity for a comparative analysis with the pre-cleaned state. The results revealed negligible changes in both transmittance and emissivity post-cleaning, suggesting that aging, rather than soiling, is the primary factor contributing to the slight reductions observed in these optical properties. The experiments in references also indicate the stability of PDMS material. The transmittance of PDMS exhibits minimal reduction (only by 0.14%) in damp-heat accelerated aging tests (1325 hours) in references 18 and 19. Furthermore, UV radiation appears to have a negligible impact on its optical properties[18, 19].

Supplementary Table 1. Type A and B uncertainties for Agilent Cary 7000 DRA and Bruker Vertex 70 acquired using their specification and acceptance tests.

**Uncertainties art**	**Type A relative uncertainties**						
Total sample position repeatability UV/Vis/NIR	0.004						
Total sample position repeatability FTIR	0.001						
Random noise	Calculated using experimental data						
	**Type B relative uncertainties**						
	**Uncertainty value in range for Cary 7000**						**FTIR Vertex 70**
	**250–600nm**	**601nm–860nm**	**860nm–1500nm**	**1.5–2.2µm**	**2.2–2.5µm**		**2.5–16µm**
Corrected baseline flatness (lamp stability)	0.0016	0.0016	0.0016	0.0016	0.0016		
Photometric Noise (RMS)	0.00012	0.00012	0.0001	0.0001	0.0001		
Photometric linearity	0.0007	0.0007	0.0015	0.0015	0.0015		
Photometric accuracy							0.001
Stray light	0.0001	0.0001	0.0001	0.0001	0.0001		
Beam polarisation	0.001	0.001	0.001	0.001	0.001		
reference standard reflection (k=2), 95% confidence	0.0053	0.0049	0.0049	0.0088	0.032		
reference port reflection value bias							0.05
**Total relative error Type B (sqrt of quadrature sum) in ordinate axis**	**0.0057**	**0.0053**	**0.0055**	**0.0091**	**0.0321**		**0.05**
Uncertainty in an abscissa (wavelength) axis	0.008 nm	0.008 nm	0.4nm	0.4 nm	0.4 nm		0.4cm^{-1}

Supplementary Movie 1.
Wettability of PMMM film. The water droplet does not stick to the PMMM and moves quickly through the surface.

Supplementary Movie 2.
Active self-cleaning via water droplets from a syringe.

Supplementary Movie 3.
Passive self-cleaning via condensed water droplets from the humidified air.

References

1. Borók, A., Laboda, K., & Bonyár, A. (2021). PDMS bonding technologies for microfluidic applications: A review. Biosensors, 11(8), 292.
2. Woolliams, Emma R. (2013). Determining the uncertainty associated with integrals of spectral quantities. Technical Report. EMRP Joint Research Project.
3. Alaee, R., Rockstuhl, C., & Fernandez-Corbaton, I. (2018). An electromagnetic multipole expansion beyond the long-wavelength approximation. Optics Communications, 407, 17-21.
4. Terekhov, P. D., Baryshnikova, K. V., Artemyev, Y. A., Karabchevsky, A., Shalin, A. S., & Evlyukhin, A. B. (2017). Multipolar response of nonspherical silicon nanoparticles in the visible and near-infrared spectral ranges. Physical Review B, 96(3), 035443.
5. Butakov, N. A., & Schuller, J. A. (2016). Designing multipolar resonances in dielectric metamaterials. Scientific Reports, 6(1), 38487.
6. Balezin, M., Baryshnikova, K. V., Kapitanova, P., & Evlyukhin, A. B. (2018). Electromagnetic properties of the Great Pyramid: First multipole resonances and energy concentration. Journal of Applied Physics, 124(3).
7. Van de Groep, J., & Polman, A. (2013). Designing dielectric resonators on substrates: Combining magnetic and electric resonances. Optics Express, 21(22), 26285-26302.
8. Spinelli, P., Verschuuren, M. A., & Polman, A. (2012). Broadband omnidirectional antireflection coating based on subwavelength surface Mie resonators. Nature Communications, 3(1), 692.
9. Chen, G., Fu, H., Zou, Y., Wang, S., Gao, Y., Yue, T., ... & Zhou, Y. (2023). A Promising Radiation Thermal Protection Coating Based on Lamellar Porous Ca‐Cr co‐Doped Y3NbO7 Ceramic. Advanced Functional Materials, 33(47), 2305650.
10. Alaee, R., Albooyeh, M., & Rockstuhl, C. (2017). Theory of metasurface based perfect absorbers. Journal of Physics D: Applied Physics, 50(50), 503002.
11. Liu, W., & Kivshar, Y. S. (2018). Generalized Kerker effects in nanophotonics and meta-optics. Optics Express, 26(10), 13085-13105.
12. Alaee, R., Filter, R., Lehr, D., Lederer, F., & Rockstuhl, C. (2015). A generalized Kerker condition for highly directive nanoantennas. Optics Letters, 40(11), 2645-2648.

13. Dezert, R., Richetti, P., & Baron, A. (2019). Complete multipolar description of reflection and transmission across a metasurface for perfect absorption of light. Optics Express, 27(19), 26317-26330.
14. Raman, A. P., Anoma, M. A., Zhu, L., Rephaeli, E., & Fan, S. (2014). Passive radiative cooling below ambient air temperature under direct sunlight. Nature, 515(7528), 540-544.
15. Mandal, J., Huang, X., & Raman, A. P. (2021). Accurately Quantifying Clear-Sky Radiative Cooling Potentials: A Temperature Correction to the Transmittance-Based Approximation. Atmosphere, 12(9), 1195.
16. Zhao, D., Aili, A., Zhai, Y., Lu, J., Kidd, D., Tan, G., ... & Yang, R. (2019). Subambient cooling of water: toward real-world applications of daytime radiative cooling. Joule, 3(1), 111-123.
17. Li, T., Heuvelink, E., Dueck, T. A., Janse, J., Gort, G., & Marcelis, L. F. M. (2014). Enhancement of crop photosynthesis by diffuse light: quantifying the contributing factors. Annals of botany, 114(1), 145-156.
18. McIntosh, K. R., Powell, N. E., Norris, A. W., Cotsell, J. N., & Ketola, B. M. (2011). The effect of damp ‐ heat and UV aging tests on the optical properties of silicone and EVA encapsulants. Progress in Photovoltaics: Research and Applications, 19(3), 294-300.
19. Xiu, Y., Zhu, L., Hess, D., & Wong, C. P. (2006, March). Superhydrophobicity and UV stability of polydimethylsiloxane/polytetrafluoroethylene (PDMS/PTFE) coatings. In 2006 11th International Symposium on Advanced Packaging Materials: Processes, Properties and Interface (pp. 98-103). IEEE.
