# Supplementary material for: Radiative cooling and indoor light management enabled by a transparent and self-cleaning polymer-based metamaterial
Source: Nat Commun. 2024 May 7;15:3798. doi: 10.1038/s41467-024-48150-2 (PMC11076518; doi:10.1038/s41467-024-48150-2)
Supplement: Supplementary file 3 — Description of Additional Supplementary Files [file 41467_2024_48150_MOESM3_ESM.pdf]

**Description of Additional Supplementary Files**

File Name: Supplementary Movie 1
Description: Wettability of PMMM film. The water droplet does not stick to the PMMM and moves quickly through the surface.

File Name: Supplementary Movie 2
Description: Active self-cleaning via water droplets from a syringe

File Name: Supplementary Movie 3
Description: Passive self-cleaning via condensed water droplets from the humidified air.
